# Mid-gestational luteal insufficiency in predisposed dog breeds: Retrospective evaluation of medroxyprogesterone acetate therapy and timed-withdrawal

**DOI:** 10.14202/vetworld.2026.2777-2786

**Published:** 2026-07-10

**Authors:** Saengtawan Arayatham, Panthipa Borikappakul, Sakchai Ruenphet

**Affiliations:** 1 Clinic of Obstetrics, Gynecology and Animal Reproduction, Faculty of Veterinary Medicine, Mahanakorn University of Technology, 140 Cheum-Sampan Road, Nong Chok, Bangkok, Thailand.; 2 Animal Biotechnology, Faculty of Veterinary Medicine, Mahanakorn University of Technology, 140 Cheum-Sampan Road, Nong Chok, Bangkok, Thailand.

**Keywords:** canine pregnancy, cesarean section, hypoluteodism, luteal insufficiency, medroxyprogesterone acetate, progesterone monitoring, Shetland Sheepdog, synthetic progestins

## Abstract

**Background and Aim:**

Hypoluteodism is an important cause of non-infectious pregnancy loss in bitches and is characterized by inadequate progesterone (P4) production by the corpus luteum. Evidence regarding breed-specific patterns of luteal insufficiency and the clinical utility of medroxyprogesterone acetate (MPA) with timed-withdrawal remains limited. This study aimed to identify the temporal pattern of luteal insufficiency in predisposed dog breeds and to evaluate the efficacy and safety of an MPA protocol combined with standardized late-gestation withdrawal.

**Materials and Methods:**

Clinical records from 29 pregnancies in 25 bitches diagnosed with hypoluteodism were retrospectively reviewed. The cohort consisted predominantly of Shetland Sheepdogs (21), together with Miniature American Shepherds (2) and Pomeranians (2). Serial serum P4 monitoring commenced during the fourth week of gestation. Hypoluteodism was diagnosed when P4 concentrations decreased below 10 ng/mL or declined by >5 ng/mL within 48–72 h. A low-dose MPA regimen (0.1 mg/kg, orally, once daily) was administered, and treatment was discontinued 24–48 h before elective cesarean section. Pregnancy outcomes and maternal and neonatal safety parameters were evaluated.

**Results:**

A distinct mid-gestational critical window was identified, with 79.3% of cases exhibiting P4 decline during weeks 5–6 of gestation. Pregnancy was successfully maintained to term in 86.2% (25/29) of pregnancies. Reproductive failure occurred in four pregnancies (13.8%), including three abortions and one case of fetal mummification. Mean litter size was approximately 4.4 puppies, yielding 110 live-born neonates, of which 94.5% survived to weaning. Female puppies showed no evidence of virilization, and no maternal complications, including diabetes mellitus, mammary hyperplasia, mammary tumors, or agalactia, were observed. Timed-withdrawal of MPA before delivery prevented premature labor and fetal compromise.

**Conclusion:**

Predisposed breeds, particularly Shetland Sheepdogs, exhibit a predictable period of luteal vulnerability during weeks 5–6 of gestation. Early P4 surveillance beginning at day 28–30 post-ovulation, combined with low-dose MPA therapy and withdrawal 24–48 h before elective delivery, represents an effective clinical strategy for reducing pregnancy loss while facilitating planned cesarean section.

## INTRODUCTION

Progesterone (P4) is essential for maintaining canine pregnancy. Unlike other domestic species, the canine placenta does not synthesize P4; therefore, the bitch relies entirely on the ovarian corpus luteum (CL) throughout gestation [1]. Consequently, premature luteolysis or functional disruption of the CL results in pregnancy failure [2, 3].

Hypoluteodism, or luteal insufficiency, is defined as a primary dysfunction in which the CL fails to secrete sufficient P4 to sustain pregnancy. This condition is typically identified by serum P4 concentrations falling below the critical threshold (commonly 2–5 ng/mL) in the absence of other abortifacient factors [4, 5]. However, in high-risk breeds or clinical cases exhibiting a precipitous decline, early intervention before reaching these critical levels may be warranted to prevent irreversible fetal loss. Although its precise incidence is underreported, largely because of challenges in establishing consistent diagnostic criteria, recent retrospective analyses indicate that hypoluteodism is a significant contributor to non-infectious pregnancy loss [6, 7]. As a result, the condition is particularly insidious, often presenting without overt clinical signs until fetal compromise has already occurred [8].

Physiologically, the regulation of the canine CL undergoes a critical transition during mid-gestation, shifting from autonomous function to reliance on hormonal support. During early gestation, the CL operates relatively independently, but beginning around days 25–30 post-ovulation, it becomes dependent on pituitary support, particularly luteinizing hormone (LH) and prolactin [9, 10]. This "critical window" of shifting luteotrophic dependency represents a period of increased vulnerability to luteal failure, as described by Kowalewski *et al.* [11]. Recent studies suggest that certain breeds may be predisposed to premature luteolysis during this transitional phase, further amplifying the risk [12]. However, distinguishing true primary hypoluteodism from secondary P4 declines associated with infectious agents, such as *Brucella canis* and *Escherichia coli*, or uterine pathology remains a diagnostic challenge for clinicians, particularly during this vulnerable interval [6].

The clinical management of confirmed hypoluteodism necessitates exogenous P4 supplementation to compensate for inadequate endogenous production. Historically, synthetic progestins, such as medroxypro-gesterone acetate (MPA), have been widely used because of their potent progestational activity and broad availability [6]. Although effective, the use of long-acting progestins remains controversial because of adverse effects, including masculinization of female fetuses, insulin resistance in the dam, and prolonged gestation with potential fetal loss when withdrawal is mistimed [13]. Although recent protocols using micronized natural P4 or altrenogest have gained popularity, MPA remains an important therapeutic option, particularly in resource-limited settings or when alternative formulations are unavailable or unsuitable. Zhelavskyi *et al.* [14] demonstrated that hormonal supplementation can effectively support pregnancy maintenance in bitches with luteal insufficiency.

Despite the recognized clinical importance of hypoluteodism, several important gaps remain in the current literature. Most available reports are limited to isolated case descriptions or small case series, with relatively few studies providing systematic longitudinal monitoring of P4 dynamics throughout gestation. In addition, previous investigations have largely involved heterogeneous canine populations, making it difficult to identify breed-specific patterns of luteal dysfunction or periods of increased susceptibility to pregnancy loss. Information regarding predisposed breeds, particularly the Shetland Sheepdog, remains scarce. Furthermore, although MPA has historically been used for pregnancy support, concerns regarding fetal virilization, prolonged gestation, and maternal adverse effects have limited its application, and contemporary evidence evaluating the safety and efficacy of low-dose MPA protocols with planned late-gestation withdrawal is limited. Consequently, there is a lack of practical clinical guidelines regarding the optimal timing for P4 surveillance, initiation of therapy, and withdrawal of exogenous progestins to facilitate elective cesarean section while minimizing fetal and maternal complications.

Therefore, this retrospective study aimed to characterize the temporal pattern of P4 decline in bitches diagnosed with hypoluteodism and to evaluate the clinical efficacy and safety of a tailored low-dose MPA protocol combined with standardized prepartum withdrawal. Particular emphasis was placed on identifying a critical period of luteal vulnerability in predisposed breeds, especially the Shetland Sheepdog, and on determining whether systematic P4 monitoring, coupled with timed cessation of treatment, could successfully maintain pregnancy to term without compromising maternal health or neonatal viability. By providing data from one of the largest documented cohorts with serial hormonal monitoring and a standardized withdrawal strategy, this study seeks to establish a practical framework for the clinical management of canine hypoluteodism and to offer veterinarians an evidence-based approach for reducing pregnancy loss while facilitating planned cesarean delivery.

## MATERIALS AND METHODS

### Ethical approval

This retrospective study was reviewed and approved by the Animal Research Ethics Committee of Mahanakorn University of Technology, Bangkok, Thailand (Approval No. ACUC-MUT-2025/012). Because this study was entirely based on the review of pre-existing clinical records and routine reproductive data, all procedures were conducted in strict accordance with institutional guidelines for animal welfare and ethical standards for veterinary clinical research. Written informed consent had been previously obtained from all owners through signed participation agreements permitting the use of their animals’ medical and reproductive data for research purposes.

### Study period and location

The study was conducted from January 2024 to December 2025 at the Clinic of Obstetrics, Gynecology and Animal Reproduction, Faculty of Veterinary Medicine, Mahanakorn University of Technology, Bangkok, Thailand. Clinical records from pregnancies managed at the referral clinic during the study period were retrospectively reviewed. Cases originated from privately owned breeding dogs that underwent routine reproductive management and pregnancy monitoring.

### Study design

This study consisted of a retrospective review of clinical records from 25 bitches comprising 29 pregnancies diagnosed with hypoluteodism. The study population included three breeds: Shetland Sheepdog (SS; n = 21; 24 pregnancies), Miniature American Shepherd (MAS; n = 2; 3 pregnancies), and Pomeranian (Pom; n = 2; 2 pregnancies). The population was heavily skewed toward SS, suggesting a potential breed predisposition.

**The mean age of the cohort was 4.2 ± 1.8 years (range:** 2–8 years), with a mean parity of 1.5 ± 0.8 previous litters. Notably, 80% (20/25) of bitches had a documented history of at least one prior unexplained pregnancy loss. Body weights ranged from 2.4 to 18.2 kg (mean 9.8 ± 2.4 kg), allowing precise calculation of the MPA dosage for each individual. Breeding management was standardized, and all bitches underwent fresh-semen artificial insemination (n = 29) following ovulation confirmation.

### Monitoring and diagnosis criteria

Ovulation timing was determined using serial P4 measurements, with ovulation defined as the point at which concentrations reached 4–10 ng/mL. After mating was confirmed, pregnancy status and fetal development were monitored using transabdominal ultrasonography [15–17].

Surveillance for hypoluteodism was conducted through weekly or biweekly serum P4 assessments beginning during the fourth week of gestation. Hypoluteodism was diagnosed based on two criteria: (1) a decline in serum P4 to <10 ng/mL or (2) a rapid decrease of >5 ng/mL within a 48–72-h interval, even when the absolute value remained slightly above the traditional threshold of 5 ng/mL.

All P4 measurements were performed using a Vcheck V200 analyzer (Bionote Co., Ltd., Hwaseong, South Korea), a validated point-of-care fluorescent immunoassay showing a high correlation with reference laboratory chemiluminescence methods [15]. Subsequent references to the analyzer are designated as Bionote.

### Therapeutic and safety protocol

When luteal insufficiency was diagnosed, pregnancy maintenance therapy was initiated using MPA (Provera®, Pfizer Inc., New York, NY, USA). Treatment commenced immediately after the detection of subthreshold P4 concentrations, as indicated by the asterisks in Table 1. A base dosage of 0.1 mg/kg once daily was administered orally, with adjustments made according to the clinical response when necessary.

During treatment, dams were monitored every 2 weeks using transabdominal ultrasonography to assess fetal heart rates (target >200 beats/min) and placental integrity. Maternal safety was evaluated through physical palpation of the mammary chains to detect mammary hyperplasia or fibroadenomas.

A key refinement of this study was the implementation of a standardized withdrawal protocol. To support appropriate fetal maturation and avoid excessively prolonged gestation, a recognized limitation of long-acting progestins, MPA treatment was discontinued 1–2 days before the planned cesarean section. The procedure was scheduled at 63 ± 1 days after ovulation, corresponding to the expected gestational length in dogs. This timing was intended to align with the pharmacokinetic profile of oral MPA in canines, thereby allowing sufficient clearance of exogenous progestins before surgical intervention.

### Monitoring of maternal and fetal safety

Following the initiation of MPA treatment, dams were monitored weekly for evidence of insulin resistance using urine glucose dipsticks and for mammary gland abnormalities. Neonatal safety was assessed immediately after cesarean section.

A systematic physical examination was performed to identify congenital abnormalities, including facial clefts, and signs of virilization in female puppies, such as clitoral hypertrophy or reduced anogenital distance. Neonatal viability was monitored until weaning at 6 weeks of age.

### Statistical analysis

Data collected included ovulation-phase P4 concentrations, timing of hypoluteodism onset, P4 concentrations at diagnosis, pregnancy outcomes (abortion versus live birth), litter size, and sex distribution. Descriptive statistical analyses were applied to characterize temporal patterns of luteal decline and evaluate the clinical effectiveness of the treatment protocol.

## RESULTS

### Demographics and onset of luteal insufficiency

A total of 29 pregnancies were monitored in this cohort. Initial luteal function appeared clinically adequate in all subjects, as reflected by post-mating serum P4 concentrations measured 1–2 days after ovulation. Values ranged from 11.23 to 19.37 ng/mL.

The onset of hypoluteodism, defined as a decline in P4 requiring exogenous P4 supplementation, varied among individuals but followed a recognizable temporal pattern. Most cases demonstrated a critical decline in P4 concentrations between weeks 5 and 6 of gestation (Table 1).

**Table 1:** The history of progesterone concentrations during mating and pregnancy, together with the schedule of MPA administration and withdrawal in female dogs.

**Case IDᵃ**	**Breed**	**Baseline P4 (ng/mL)** ** Ovulation**	**Baseline P4 (ng/mL)** ** 1ˢᵗ mating**	**Diagnosis of ** **hypoluteodism** **ᵇ** ** Onset week**	**Diagnosis of ** **hypoluteodism** **ᵇ** ** P4 level (ng/mL)**	**Management** ** MPA withdrawalᶜ**	**Outcome**	**Neonatal defects/** **virilization**
D1	SS	N/A	17.00	6	7.86	−2 days	Live birth (3M, 3F)	None
D2	SS	N/A	19.37	5	7.34	−1 day	Live birth (4M)	None
D3	SS	7.83	N/A	6	8.68	−2 days	Live birth (3M, 6F)	None
D4	SS	7.66	N/A	6	4.43	–	Abortion at Wk 8	N/A
D5-Aᵈ	SS	8.31	N/A	5	8.70	–	Abortion at Wk 8	N/A
D5-Bᵈ	SS	6.32	N/A	5	7.80	–	Abortion at Wk 8	N/A
D5-Cᵈ	SS	8.95	N/A	4	7.43	−1 day	Four mummified fetuses	None (mummified)
D6	SS	7.55	N/A	6	8.62	−2 days	Live birth (2M, 3F)	None
D7	SS	5.18	N/A	7	5.40	−2 days	Live birth (2M, 1F)	None
D8	SS	N/A	12.04	7	5.07	−2 days	Live birth (3M, 1F)	None
D9	SS	7.51	12.08	5	7.06	−1 day	Live birth (2M, 2F)	None
D10-Aᵈ	MAS	N/A	12.54	5	8.80	−1 day	Live birth (3M, 3F)	None
D10-Bᵈ	MAS	N/A	12.20	7	5.65	−2 days	Live birth (3M, 4F)	None
D10-Cᵈ	MAS	10.50	N/A	5	6.53	−2 days	Live birth (3M, 2F)	None
D11	SS	6.98	N/A	5	8.70	−1 day	Live birth (2M, 1F)	None
D12	SS	N/A	13.32	5	7.70	−1 day	Live birth (4F)	None
D13	SS	9.14	N/A	6	9.91	−2 days	Live birth (4M)	None
D14	SS	6.75	N/A	5	8.12	−1 day	Live birth (4M, 2F)	None
D15	SS	N/A	14.20	5	8.05	−1 day	Live birth (2F)	None
D16	SS	8.76	N/A	5	7.05	−2 days	Live birth (3M, 2F)	None
D17	SS	8.13	N/A	5	9.87	−1 day	Live birth (2M, 1F)	None
D18	Pom	7.67	N/A	5	7.85	−2 days	Live birth (1M)	None
D19	SS	N/A	11.79	7	5.35	−2 days	Live birth (3M, 3F)	None
D20	Pom	5.09	N/A	6	8.51	−2 days	Live birth (1M, 1F)	None
D21	SS	10.71	N/A	6	8.90	−2 days	Live birth (4M, 1F)	None
D22	SS	7.53	N/A	4	7.85	−2 days	Live birth (1M)	None
D23	MAS	N/A	12.55	5	9.70	−1 day	Live birth (3M, 1F)	None
D24	SS	9.41	N/A	5	8.52	−2 days	Live birth (3M, 2F)	None
D25	SS	N/A	11.23	5	7.57	−1 day	Live birth (4M, 2F)	None

ᵃCase ID = Unique identifier for each pregnancy event. ᵇDiagnosis = Defined as the first gestational week in which serum P4 decreased below the threshold (<10 ng/mL), necessitating MPA initiation. ᶜMPA withdrawal = Timing of MPA cessation relative to the scheduled elective cesarean section. ᵈIndicates distinct pregnancies occurring in the same bitch.

All live-born puppies (n = 110) were examined at birth and again at 6 weeks of age (n = 104). No congenital abnormalities or virilization were detected in female puppies.

F = Female; M = Male; MAS = Miniature American Shepherd; MPA = Medroxyprogesterone acetate; N/A = Not applicable; P4 = Progesterone; Pom = Pomeranian; SS = Shetland Sheepdog.

Among the monitored pregnancies, luteal insufficiency was detected in 6.9% (2/29) of cases during week 4. The highest incidence occurred during week 5, affecting 55.2% (16/29) of pregnancies, followed by week 6, in which 24.1% (7/29) of cases required treatment. Late-onset luteal insufficiency during week 7 was observed in 13.8% (4/29) of pregnancies. Overall, most cases (79.3%) exhibited a critical decline in P4 concentrations between weeks 5 and 6 of gestation, demonstrating a highly consistent temporal pattern of luteal failure.

### Treatment efficacy and pregnancy outcomes

The MPA protocol maintained pregnancy to term in 86.2% (25/29) of cases. Longitudinal P4 profiles showed a mean decline from 15.3 ± 2.1 ng/mL at week 4 to 7.8 ± 1.2 ng/mL at the time of diagnosis during weeks 5–6. All successful pregnancies resulted in healthy puppies delivered by elective cesarean section according to standardized obstetrical protocols.

Litter characteristics showed considerable variability, with litter sizes ranging from 1 to 9 puppies and an average of approximately 4.4 puppies. The sex distribution was male-biased, comprising 63 M and 47 F.

Notably, the planned withdrawal of MPA 1–2 days before elective cesarean section in all 25 successful pregnancies resulted in the absence of premature labor or fetal compromise. These findings provide systematic evidence supporting the safety and effectiveness of this timed-withdrawal approach in a relatively large cohort.

### Reproductive failures

Reproductive failure was documented in 13.8% (4/29) of the monitored pregnancies despite routine P4 surveillance and timely clinical intervention.

**Abortion:** Three pregnancies (subjects D4, D5-A, and D5-B) resulted in abortion. In these cases, serum P4 concentrations declined to 4.43, 8.70, and 7.80 ng/mL during weeks 6, 5, and 5 of gestation, respectively, coinciding with pregnancy loss.

**Fetal mummification:** Subject D5-C experienced an additional reproductive failure characterized by four mummified fetuses. This outcome occurred despite the early initiation of MPA treatment during week 4, when the P4 concentration had decreased to 7.43 ng/mL.

**Neonatal outcomes and maternal safety:** Among the 25 successful pregnancies, a total of 110 puppies (63 M and 47 F) were born with birth weights consistent with breed standards, and 94.5% (104/110) survived until weaning. No maternal complications, including clinical diabetes mellitus, mammary hyperplasia, or lactation failure (agalactia), were documented, indicating overall reproductive success.

However, one subject (D5-C) experienced fetal mummification during week 4. Although this event could not be definitively attributed to MPA administration, it may have reflected an unfavorable uterine environment.

In addition to survival outcomes, all neonates were assessed using a modified Apgar score at 0 and 5 min after delivery. Overall, 92% of puppies achieved scores >7, indicating satisfactory neonatal health. Mean birth weights were consistent with breed standards, with SS puppies weighing 185 ± 25 g on average.

Detailed perineal examinations of the 47 female puppies revealed no clitoral hypertrophy, reduced anogenital distance, or other signs of virilization. Follow-up evaluations until weaning at 6 weeks of age showed no evidence of MPA-associated congenital abnormalities, including facial clefts, as reported in previous high-dose studies.

Maternal follow-up revealed that all successful dams resumed normal estrous cycles within 5–7 months after whelping. Furthermore, no cases of secondary insulin resistance or mammary tumors were observed during the 6-month postpartum period.

## DISCUSSION

The findings of this retrospective study underscore the critical importance of serial P4 monitoring during the second trimester of canine pregnancy. Our data demonstrate that hypoluteodism is not a stochastic event but rather emerges consistently within a critical window between weeks 5 and 6 of gestation.

### Temporal pattern of luteal insufficiency and the critical window

In this cohort, 79.3% of pregnancies required exogenous P4 supplementation during weeks 5–6. This observation corresponds to the well-characterized physiological transition of the canine CL from LH independence to combined LH and prolactin dependence during mid-gestation [18, 19]. Recent large-scale evaluations of P4 dynamics in pregnant bitches similarly reported that, although a gradual decline in serum P4 is expected, abrupt reductions below 5–10 ng/mL during this stage are indicative of luteal insufficiency and are strongly associated with pregnancy loss [20]. Complementary findings by Hinderer *et al. *[4] suggest that, although some individuals may sustain pregnancy at lower concentrations, values below these thresholds, particularly in genetically predisposed breeds, necessitate prompt intervention to prevent embryonic resorption.

The high incidence of luteal failure observed in the SS population during this period reinforces the possibility of breed-specific susceptibility to premature luteolysis or impaired prolactin-mediated luteotrophic signaling. To our knowledge, this study is among the first to quantify a narrow and predictable period of vulnerability (weeks 5–6) in SS. Although a gradual decline in P4 is expected across breeds, the clustering of 79.3% of cases within this interval highlights a breed-specific susceptibility during the transition from LH independence to hormonal dependence. From a clinical perspective, this finding supports targeted hormonal surveillance beginning at days 28–30 post-ovulation in predisposed breeds.

### Justification of the 10 ng/mL threshold

The diagnostic threshold of <10 ng/mL used in the present study is more conservative than the 2–5 ng/mL range commonly cited for pregnancy maintenance. However, this threshold was selected because the study population consisted predominantly of high-risk SS with a history of previous pregnancy loss. By intervening immediately after detecting a rapid decline in P4, the objective was to prevent concentrations from reaching levels associated with irreversible placental separation or embryonic resorption.

Although this approach may have resulted in treatment of some bitches that could have maintained pregnancy at lower P4 concentrations, it provided an additional safety margin for breeds predisposed to abrupt luteal failure. Therefore, the threshold adopted in this study should be interpreted as a proactive clinical strategy rather than a strict physiological cutoff.

### Efficacy of MPA supplementation

The MPA protocol achieved an 86.2% success rate in maintaining pregnancies to term, which is consistent with previous reports demonstrating the effectiveness of progestin supplementation in managing luteal insufficiency [20]. Administration of MPA effectively compensated for inadequate endogenous P4 production, thereby suppressing premature myometrial activity and maintaining endometrial quiescence.

One of the principal concerns associated with synthetic progestins is the risk of prolonged gestation and fetal compromise. Nevertheless, findings from the present cohort indicate that withdrawal of MPA 24–48 h before the scheduled aesarean section was both safe and effective. This interval appeared sufficient to allow clearance of the exogenous progestin and restoration of parturition mechanisms while preserving fetal viability, consistent with current clinical recommendations [6].

Furthermore, Zhelavskyi *et al.* [14] emphasized that maintaining physiologically adequate P4 concentrations is the primary determinant of implantation success and fetal survival in bitches with luteal insufficiency. These findings further support the clinical rationale for MPA supplementation in pregnancies at risk of luteal failure.

### Clinical rationale for low-dose MPA and the treatment threshold

The absence of virilization among the 47 F puppies contrasts with historical concerns regarding synthetic progestins. This favorable outcome may be attributed to the low dosage used (0.1 mg/kg) and the planned withdrawal of treatment 24–48 h before aesarean section. In addition, elective surgery performed according to fetal maturity, rather than waiting for spontaneous labor, likely minimized the risk of MPA-induced dystocia or prolonged gestation.

However, safety conclusions should be interpreted with caution due to the retrospective design and the absence of an untreated control group. Therefore, the present protocol is more appropriately described as clinically effective under intensive monitoring rather than universally safe.

Although micronized P4 and altrenogest are often preferred because of their shorter half-lives, MPA remains a potent and economical alternative. Importantly, the risk of fetal masculinization associated with synthetic progestins is dose dependent. The use of a low-dose regimen and avoidance of exposure during early organogenesis before day 30 probably contributed to the absence of developmental abnormalities observed in this study. Moreover, the conservative threshold of 10 ng/mL provided a practical clinical buffer for breeds such as SS, in which luteal collapse may occur more abruptly than in mixed-breed populations.

### Refractory hypoluteodism and recurrent pregnancy loss

Despite the high overall success rate, the recurrent reproductive failure observed in subject D5 highlights the multifactorial nature of canine pregnancy loss. This bitch experienced pregnancy loss during three consecutive gestations, initially through abortion and subsequently through fetal mummification, despite receiving the same therapeutic protocol that was successful in other animals. These findings suggest refractory hypoluteodism or the presence of concurrent pathological conditions that adversely affected pregnancy maintenance.

Mantziaras and Zakosek Pipan [6] emphasized that pregnancy loss in the bitch is often multifactorial and extends beyond endocrine dysfunction to include infectious agents such as *B. canis*, *E. coli*, and *Salmonella* spp., as well as uterine disorders including cystic endometrial hyperplasia and chromosomal abnormalities. Similarly, the occurrence of fetal mummification during the third pregnancy of subject D5 suggests an unfavorable uterine environment or placental insufficiency rather than isolated luteal dysfunction [8, 21].

Therefore, in cases of recurrent reproductive failure despite adequate P4 supplementation, comprehensive diagnostic evaluation for infectious diseases and uterine pathology is strongly recommended [6].

### Limitations of the study

An important point of discussion concerns the use of a treatment threshold of <10 ng/mL, which exceeds the traditionally accepted range of 2–5 ng/mL required to maintain pregnancy. This proactive threshold was based not only on the absolute P4 concentration but also on the rate of decline. A rapid decrease from >15 ng/mL to <10 ng/mL over a short period was interpreted as evidence of impending luteal failure. Given the high-risk nature of the cohort, particularly the predominance of SS, early supplementation was initiated to prevent P4 concentrations from reaching levels associated with embryonic resorption.

It is possible that some bitches could have maintained pregnancy at concentrations between 5 and 10 ng/mL without intervention. Consequently, the reported success rate should be interpreted in the context of this preemptive therapeutic strategy. Because the study was retrospective and uncontrolled, the true efficacy of the protocol cannot be conclusively determined.

In clinical practice, withholding treatment from bitches suspected of hypoluteodism presents ethical challenges. Therefore, the study was necessarily observational and descriptive rather than experimentally controlled.

Another limitation was the marked predominance of SS in the study population (21/25 animals). This breed may have an inherent predisposition to luteal insufficiency or exhibit heightened sensitivity during the mid-gestational transition, potentially influencing the observed hormonal patterns. Consequently, the present findings, particularly those concerning the timing and rate of P4 decline, may be most representative of SS, and extrapolation to other breeds should be undertaken cautiously.

The lack of a nontreated control group remains a major limitation. However, considering the history of previous pregnancy loss in a substantial proportion of the cohort, withholding treatment was considered ethically unacceptable by both owners and the institutional ethics committee. Furthermore, the skewed breed distribution indicates that, although the findings are highly relevant to SS, caution is warranted when extending the identified week 5–6 critical window to giant breeds or breeds with substantially different metabolic characteristics.

### Clinical implications of the MPA withdrawal protocol

A notable contribution of the present study is the demonstration that short-term withdrawal of MPA before aesarean section (24–48 h) permits endogenous hormonal recovery without compromising fetal viability. Previous reports have highlighted concerns regarding the risk of prolonged gestation associated with MPA administration; however, the standardized withdrawal strategy described herein provides a practical framework for managing elective aesarean section.

These findings support the continued use of MPA as a viable and effective therapeutic option, particularly in resource-limited settings, when combined with intensive monitoring and appropriately timed-withdrawal.

**Figure 1:**
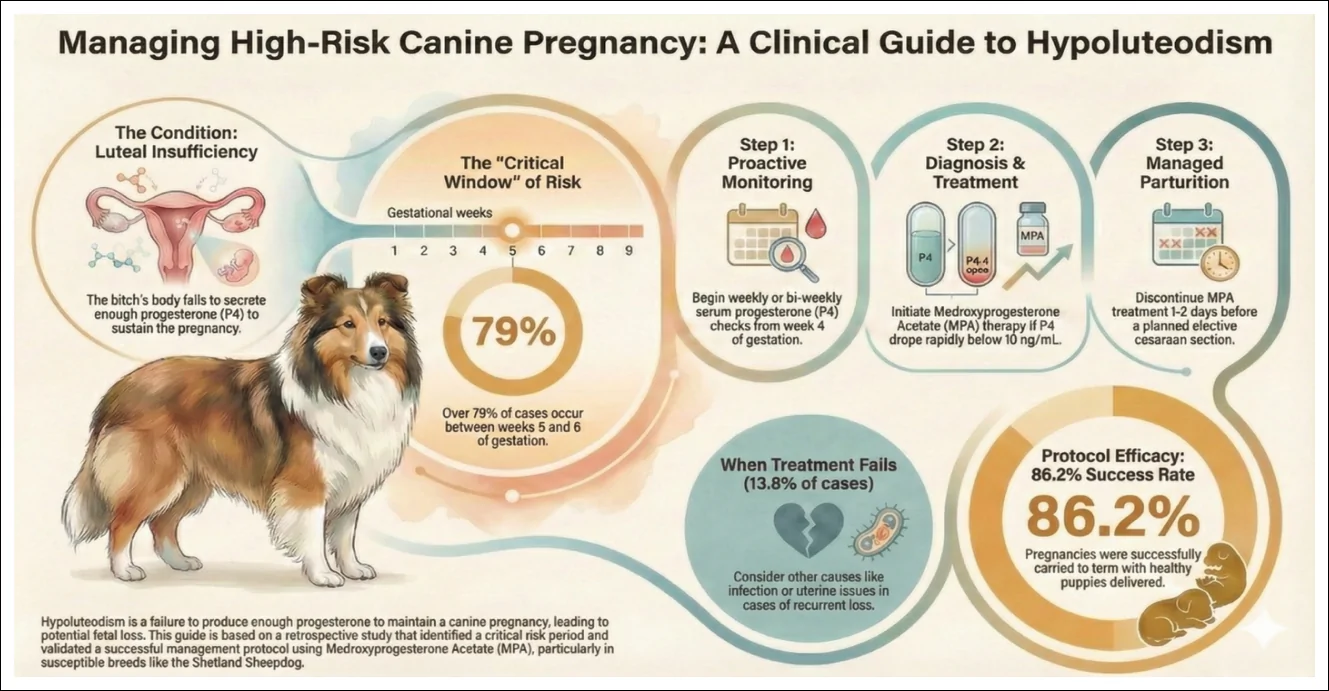
Infographic illustrating the management of high-risk canine pregnancy as a clinical guide for hypoluteodism.

In addition, the present study includes an infographic summarizing the clinical management of canine hypoluteodism (Figure 1). This visual representation outlines the pathophysiology of the condition, the critical gestational window for onset (weeks 5–6), diagnostic criteria, the MPA treatment protocol with planned withdrawal, and the overall reproductive outcomes observed in the cohort.

## CONCLUSION

This retrospective study demonstrated that hypoluteodism in predisposed dog breeds, particularly SS, follows a consistent temporal pattern, with 79.3% of cases developing luteal insufficiency during weeks 5–6 of gestation. Implementation of serial P4 monitoring and timely intervention with a low-dose MPA protocol resulted in successful maintenance of pregnancy to term in 86.2% of cases. Among the 25 successful pregnancies, 110 live puppies were delivered, of which 94.5% survived to weaning. Importantly, no evidence of virilization in female offspring, premature labor, maternal diabetes mellitus, mammary hyperplasia, mammary tumors, or lactation failure was observed, supporting the favorable maternal and neonatal outcomes associated with the treatment strategy.

A major strength of this study is that it represents one of the largest documented cohorts of bitches with hypoluteodism managed using systematic serial hormonal monitoring and a standardized timed-withdrawal protocol. Furthermore, the study provides novel evidence supporting the existence of a breed-associated critical period for luteal failure in SS and demonstrates the feasibility of using a low-dose MPA regimen under intensive clinical monitoring.

Future prospective controlled studies involving larger and more diverse canine populations are warranted to validate the proposed critical window, investigate the underlying mechanisms responsible for breed susceptibility, and compare MPA with alternative progestin therapies. Further research evaluating long-term reproductive outcomes and offspring development would also provide valuable information regarding the safety of hormonal intervention.

Overall, the present findings indicate that hypoluteodism in predisposed breeds is characterized by a predictable period of luteal vulnerability during mid-gestation and that a carefully monitored low-dose MPA protocol with strategic prepartum withdrawal represents an effective and practical approach for preserving pregnancy and optimizing reproductive outcomes. These results provide clinicians with an evidence-based strategy for managing canine luteal insufficiency and contribute to the refinement of reproductive medicine in dogs.

## DATA AVAILABILITY

The data generated during the study are included in the manuscript.

## GENERATIVE AI DECLARATION

The authors used generative artificial intelligence tools to assist with language editing and image generation. The generated content was critically reviewed, revised, and verified by the authors. All experimental design, data collection, analysis, interpretation, and conclusions were performed by the authors, who assume full responsibility for the content of the article. No artificial intelligence tool was credited as an author.

## AUTHORS’ CONTRIBUTIONS

SA, PB, and SR: Conceptualization, formal analysis, methodology, writing – original draft, and writing – review and editing. SR: Investigation and supervision. SA and SR: Project administration. All authors have read and approved the final version of the manuscript.
